# Crystal Structures, Hirshfeld Surfaces, and Thermal Study of Isostructural Polymeric Ladders of La(III) and Sm(III) Coordination Compounds with 4,4’-Bipyridine and Dibromoacetates

**DOI:** 10.3390/ma13194274

**Published:** 2020-09-25

**Authors:** Agnieszka Czylkowska, Anna Pietrzak, Małgorzata Szczesio, Bartłomiej Rogalewicz, Jakub Wojciechowski

**Affiliations:** Institute of General and Ecological Chemistry, Faculty of Chemistry, Lodz University of Technology, Zeromskiego 116, 90-924 Lodz, Poland; anna.pietrzak.1@p.lodz.pl (A.P.); malgorzata.szczesio@p.lodz.pl (M.S.); 211150@edu.p.lodz.pl (B.R.); j.m.wojciechowski@gmail.com (J.W.)

**Keywords:** coordination polymer, lanthanum and samarium complexes, TG-MS study, X-ray structure, Hirshfeld surface, enrichment ratio

## Abstract

Two novel mixed ligand complexes with general formula [M_2_(4,4′-bpy)_1.5_(CBr_2_HCOO)_6_(H_2_O)_2_]_n_ (where 4,4′-bpy = 4,4′-bipyridine) were synthesized. Thermal analysis was used to describe a solid intermediate and final products of thermolysis. A coupled TG-MS system was used to monitor principal volatile fragments evolved during pyrolysis. Crystal structures of the complexes were determined. Cationic dinuclear M_2_ (M(III) = La, Sm) coordination cores were obtained. Both crystal structures are isostructural. Single crystal X-ray diffraction analysis revealed that investigated structures of 1D coordination polymers assembled in ladder-like systems. The central atom replacement resulted in unit cell identity parameter П = 0.0091. Additionally, the isostructurality of the reported La(III) and Sm(III) complexes was revealed using Hirshfeld Surface analysis supported by Enrichment Ratio calculations.

## 1. Introduction

Coordination polymers are compounds that comprise an interesting and promising field of chemistry. One of the reasons is their valuable and oftentimes unusual properties. These compounds find applications in many different areas of everyday life, industry, and science. Some of the examples are sensors and biosensors [1,2,3,4,5,6,7,8], electronic and optoelectronic devices [9,10], chemical catalysts, and photocatalysts [11,12,13,14,15,16]. Some coordination polymers can be used to remove harmful substances [17,18,19,20] or to capture and entrap other molecules [21,22]. It is one of the promising ideas of dealing with the increasing levels of carbon dioxide in the air [23] and can also be applied in modern medicine, e.g., cancer therapy [24]. Coordination polymers can form one-, two-, or three-dimensional structures. Both metal-organic structures and typical Werner complexes can form polymeric chains. The formation of different polymeric structures results in changes of physical and chemical properties of stand-alone complexes and ligands and, thus, allows for their improvement and optimization. As a result, these compounds are the center of many researchers’ attention. 4,4′-Bipyridine is a type of ligand that is often used in the synthesis to obtain coordination polymers. Such complexes exhibit very unique properties like photoluminescence or photochromism [25,26,27,28], biological [29,30] and magnetic properties [26,31], or the ability to detect or absorb harmful substances [32,33,34,35]. In this paper, we present synthesis, thermal properties, and crystal structures of two 4,4′-bipyridine coordination polymers with lanthanum (III) and samarium (III) dibromoacetates. Our previous papers have covered crystal structures of similar compounds [36,37,38]. Both ligands that we used are unique chemical compounds due to their possible ways of forming coordination bonds towards metal ions. 4,4′-Bipyridine is one of the isomers of bipyridine. This molecule may coordinate as a bridging N-donor ligand, thus, increasing the possibility of obtaining a coordination polymer. Another ligand coordinates through the carboxylate group, which can bind to metal ions in different ways and also may create polymeric structures [36,37,38]. This work is a continuation of our research concerning lanthanide coordination polymers with selected N-donor and O-donor ligands.

## 2. Materials and Methods

### 2.1. Materials

4,4′-Bipyridine, CBr_2_HCOOH, La_2_O_3_, and Sm_2_O_3_ were obtained from Sigma Aldrich, St. Louis, MO, USA.

Yellow, single crystals of investigated coordination polymers were obtained at room temperature after several weeks of slow evaporation of La(4,4′-bpy)(CBr_2_HCOO)_3_⋅H_2_O [39] and Sm(4,4′-bpy)_0.5_(CBr_2_HCOO)_3_⋅2H_2_O [40] filtrates. Both compounds were washed with 40% *v/v* ethanol and then with ethanol and diethyl ether mixture (1:1). Next, they were dried in open air. The coordination polymers were characterized by elemental analysis, thermal analysis, single-crystal X-ray diffraction, and Hirshfeld Surface calculations.

C_27_H_22_Br_12_N_3_O_14_La_2_ (1849.10 g/mol), yield (2.13%), Analytical Calculated: C, 17.54, H, 1.20, N, 2.28. Found: C, 17.36, H, 1.17, N, 2.52.

C_27_H_22_Br_12_N_3_O_14_Sm_2_ (1872.01 g/mol), yield (1.99%), Analytical Calculated: C, 17.32, H, 1.19, N, 2.25. Found: C, 17.45, H, 1.21, N, 2.46.

### 2.2. Methods

The contents of carbon, hydrogen, and nitrogen were determined by a Vario micro company Elementar Analysensysteme GmbH (Langenselbold, Germany). The TG-MS coupled measurements were performed out for La(III) and Sm(III) complexes using the Netzsch TG 209 apparatus (Selb, Germany) coupled with Netzsch MS spectrometer (Selb, Germany) in the temperature range 25–1000 °C at a heating rate of 10 °C·min^−1^, in flowing dynamic air atmosphere v = 20 mL·min^−1^ using ceramic crucibles. As a reference material, ceramic crucibles were used.

In single crystal X-ray analysis, the crystals formed yellow plates. The intensity data was collected on a Kuma CCD diffractometer. Crystal structure refinement was carried out with SHELX [41,42]. Crystallographic information files for the crystal structures are available under deposition numbers 2021532 and 2021533 (Supplementary Materials).

Hirshfeld Surface (HS) and Fingerprint (FP) analysis. The CrystalExplorer 17.5 software was used to generate Hirshfeld Surfaces [43,44]. Molecular geometries were derived from the crystal structures. For molecular fragments with the crystallographic disorder identified, only the major components were considered. The HSs for analysis were generated for asymmetric units of polymeric systems. Respective parameters, i.e., distances from the HS to the nearest atom interior (*d_i_*) and exterior (*d_e_*) to the surface are plotted as scattergrams, namely Fingerprints (FPs). A quantitative decomposition analysis of atom-to-surface contacts was calculated as a percentage of the points in the Hirshfeld Surface with *d_i_* and *d_e_* for specific atomic pairs. Additionally, the analysis is supported by Enrichment Ratio (ER) calculations for meaningful contacts between atomic pairs [45].

## 3. Results and Discussion

### 3.1. Thermal Study

Thermal analysis was used to describe pyrolysis in air atmosphere of lanthanum (III) and samarium (III) coordination compounds (Table 1). Solid intermediate and final products of decompositions of polymers were determined on the basis of the mass losses and volatile products of thermolysis. Both complexes are stable up to 50 °C. The first step of decomposition is dehydration. The complexes lose water molecules in the temperature ranges: 50–135 °C for La(III) and 50–125 °C in the case of the Sm(III) compound. Anhydrous species are stable up to 200 and 195 °C, respectively. When the temperature rises on TG curves, mass loss is observed due to the degradation of the bridging organic ligands (one molecule of 4,4′-bipyridine and four dibromoacetates). The mass losses found on TG curves are in good agreement with the calculated ones. These processes occur for La(III) and Sm(III) compounds in the ranges ca. 200–340 and 195–325 °C, respectively. Further heating causes several overlapping steps associated with the total decomposition of ligands. The horizontal mass level for La_2_O_3_ begins at 950 °C (found 17.0%, calc. 17.62%) and, for Sm_2_O_3_, begins at 945 °C (found 19.0%, calc. 18.63%).

Coordination polymers of La(III) and Sm(III) are characterized by the lowest thermal stability compared to the type of complexes published in References [39,40]. This is likely due to their structures. It also has an impact on temperatures at which the final solid decomposition products are formed. These temperatures are higher for the coordination polymers when compared to the complexes of La(III) and Sm(III) described in References [39,40].

### 3.2. TG-MS Spectra

**Figure 1:**
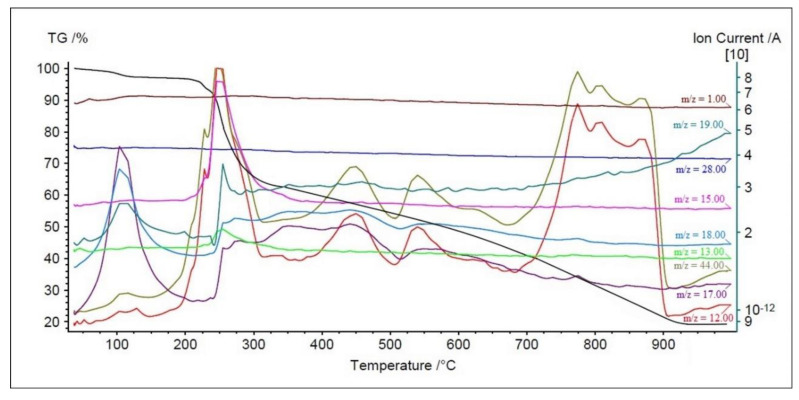
TG curve for Sm(III) complex and ion current detected by MS spectra for several mass fragments.

The TG–MS system was used to analyze principal volatile thermal decomposition and fragmentation products evolved during pyrolysis of La(III) and Sm(III) complexes in air. Their TG-MS spectra are very similar. The principal mass fragments correspond to: OH^+^, H_2_O^+^, CO_2_^+^, C^+^, Br^+^, HBr^+^, and others. For La(III) and Sm(III) compounds, major maxima for ion currents are observed in the temperature range of 200–300 °C. The first peaks for OH^+^ and H_2_O^+^ (m/z = 17, 18) occur at around 100 °C and are connected with the dehydration of the complexes. Next, OH^+^ and H_2_O^+^ are produced by oxidation of organic ligands (peaks at 250, 350, 450, and 550 °C). The profiles of C^+^ and CO_2_^+^ (m/z = 12, 44) exhibit maxima at 210, 240, 450, and 530 and a very broad one between 780–880 °C. The profiles of CH_2_O^+^ or NO^+^ (m/z = 30) appear at about 250 °C. The major ion signals containing bromide: Br^+^, HBr^+^, and CBrH^+^ have one center in the temperature range of 235 to 300 °C. The strong peak of NH^+^ (m/z = 15) was monitored at 250 °C. The mass spectrometer detected trace amounts of other fragments. The data above suggests a simultaneous decomposition of both 4,4′-bipyridine and dibromoacetate ions. The rise in temperature (above 400 °C) causes burning of the organic residues and the formation of final solid thermal decomposition products (La_2_O_3_ and Sm_2_O_3_, respectively) at about 950 °C. Figure 1, as an example, presents the correlation of some ion currents on the TG curve of the Sm(III) complex in the atmosphere.

### 3.3. Crystal Structure Determination

**Figure 2:**
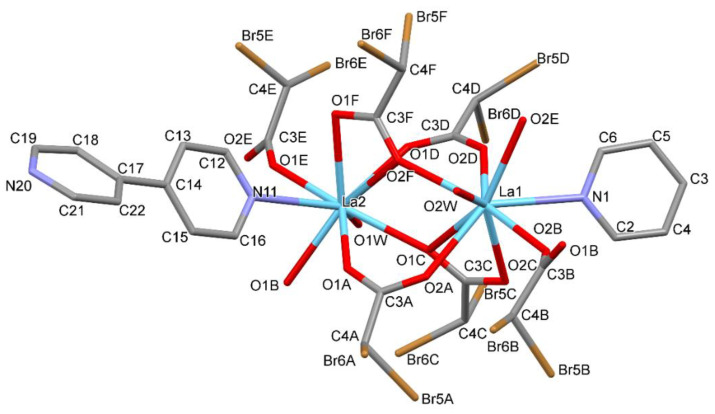
The molecular structure of the complex with La(III) (compound 1) showing the atom-labelling schemes.

**Figure 3:**
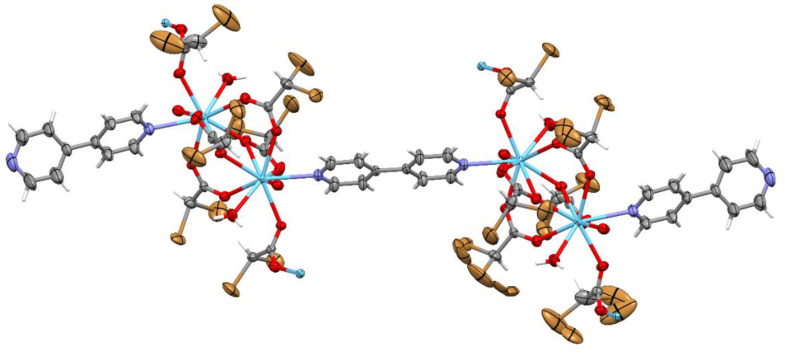
The molecular structure of complex with La(III) (compound 1), the displacement ellipsoids are drawn at the 50% probability level except H atoms.

**Figure 4:**
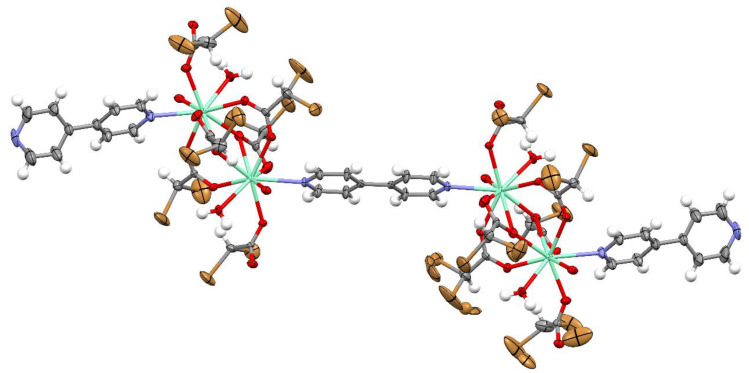
The molecular structure of the complex with Sm(III) (compound 2), the displacement ellipsoids are drawn at the 50% probability level except H atoms.

Complexes 1 and 2 crystallize in the triclinic space group *P*-1 with the same coordination spheres with formula [M_2_(4,4′-bpy)_1.5_(CBr_2_HCOO)_6_(H_2_O)_2_]_n_ (M(III) = La, Sm). Crystal data and structure refinement details are summarized in Table 2. The numbering of atoms is shown in Figure 2. The cationic dinuclear M_2_ (M(III) = La, Sm) coordination cores were obtained. Both crystal structures are isostructural with approximately the same lattice parameters. The central atom replacement resulted in unit cell identity parameter П = 0.0091 [46]. The asymmetric unit consists of one and a half molecules of bipyridine, two water molecules, six dibromoacetates, and two cations (Figure 2). Three dibromo-acetate molecules in both examined structures show a disorder (Figure 3 and Figure 4).

**Figure 5:**
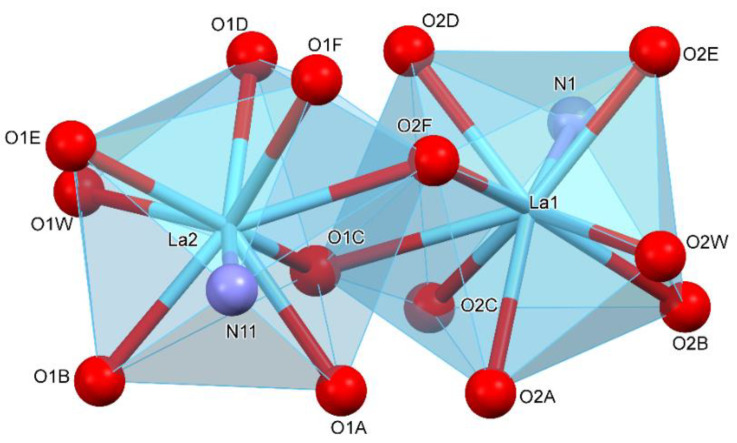
The lanthanum coordinate polyhedron.

**Figure 6:**
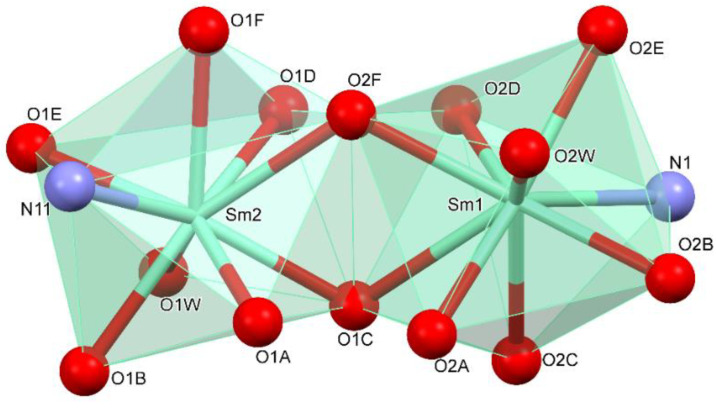
The samarium coordinate polyhedron.

**Figure 7:**
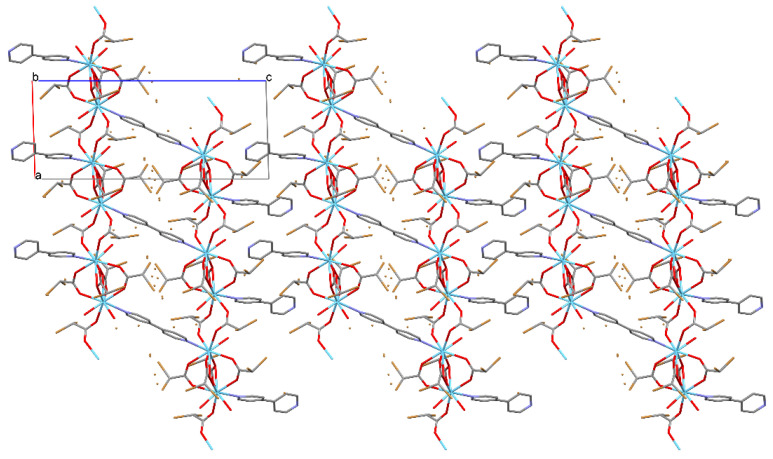
Packing of the molecules in the crystal of compound 1 along the *b* axis. The orange dots are disordered sites of Br atoms.

The coordination polyhedral can be described as a trigonal prismatic, square face tricap (Figure 5 and Figure 6). Both metals are nine coordinate. The central atoms are coordinated by three oxygen atoms from chelating-bridging, tridentate dibromoacetate ligands, four oxygen atoms from bridging dibromoacetate substituents, one water oxygen atom, and one nitrogen atom from 4,4’-bipyridine. The cores are linked together by two common oxygen atoms from tridentate dibromoacetate ligands and two O–C–O bridges formed by carboxylate groups of dibromoacetates. The metal cores and dibromoacetic acid form a one-dimensional polymeric chain. Two of these are bounded together by 4,4’-bipyridine, which has an inversion point lying on the midpoint of the bond linking two pyridine rings. The other 4,4’-bipyridine molecule coordinates metal (III) only with one nitrogen atom. The second nitrogen atom is involved in hydrogen bonding with a water molecule inside the coordination sphere of the second core metal, which creates a two-dimensional supramolecular structure (Figure 7). The layers of polymeric chains are connected by weak Br^...^Br and Br^...^H–C interactions.

Two known analogous complexes that differ in the ligand contain dichloroacetates [37,38]. These structures adopt a polymer arrangement. The biggest difference is the cationic dinuclear coordination cores for the currently studied complexes. Additionally, the lanthanum atom is ten coordinate and the samarium atom is nine coordinate for previous structures.

**Figure 8:**
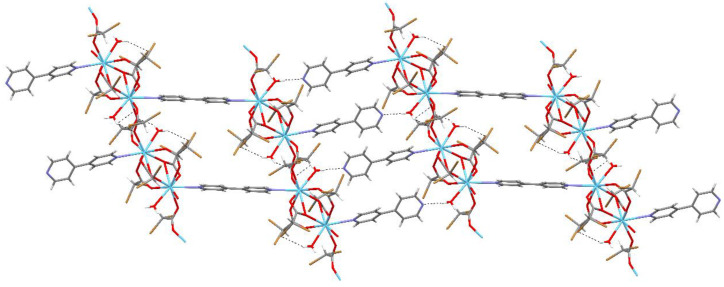
The intermolecular hydrogen bonds for compound 1.

Strong hydrogen bonds are formed by water molecules (Table 3 and Table 4). The O1W water forms ring hydrogen bonds that stabilize the coordination sphere. In the case of the O2W water molecule, the O2W—H2WA···O1E hydrogen bond system forms ring R(6) (according to the definition of Bernstein et al. [47]), which stabilizes the coordination sphere. Another O2W—H2WB···N20 hydrogen bond forms infinite chain C(22) connecting neighboring molecules through a bipyridine (Figure 8). The compound with Sm(III) creates hydrogen bonds analogous to that of compound 1. Additionally, there are weak C–H^...^O and C–H^...^Br interactions that stabilize the polymer system.

### 3.4. Hirshfeld Surface Analysis

The Hirshfeld Surface analysis provides insight into the neighborhood of the molecules or molecular fragments within the crystalline environment. Therefore, as a complementary tool for traditional structural descriptions, HS and FP are widely used for crystal packing comparisons [48,49]. Originally, the methodology was dedicated to the structures formed by hydrogen bonded molecules or discrete complexes. However, the growing interest in crystal engineering and supramolecular chemistry leads to the continuous development of their applicability. Since the analysis of polymeric structures has to be conducted for a molecular fragment only, we should be aware of the fact that it would represent not only intermolecular contacts but also covalent bonding being intersected by the generated HS. The HS analysis supported by an Enrichment Ratio (ER) calculation allows evaluating the propensity of studied systems to form particular interactions. Favored contacts, i.e., with *ER_XY_* higher than unity, have a high propensity to form contacts, while element pairs that tend to avoid contacts are characterized by *ER_XY_* < 1.

**Figure 9:**
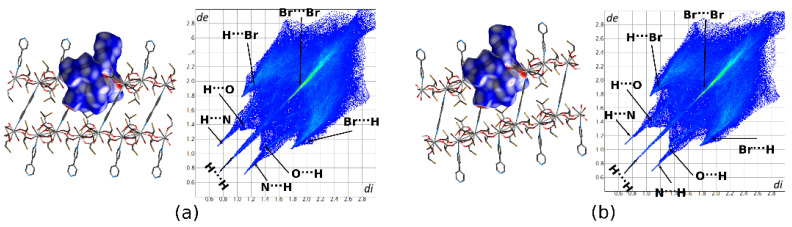
Hirshfeld Surfaces and 2D Fingerprint Plots (FPs) generated for the asymmetric unit of 1 (**a**) and 2 (**b**). Over the HS d_norm_ is mapped. d_norm_ is visualized over a fixed color scale of −1.12 (red), 0.47 (white), to 1.46 (blue). d_e_ and d_i_ are the distances to the nearest atomic exterior and interior to the surface.

Both structures 1 and 2 were characterized using 3-D Hirshfeld Surface and deriving two dimensional FP maps (Figure 9). Relevant HSs were generated for the asymmetric units of the structures. Perfectly matching FPs and almost identical percentage contributions of major intermolecular contacts confirm the isostructurality of both systems.

**Figure 10:**
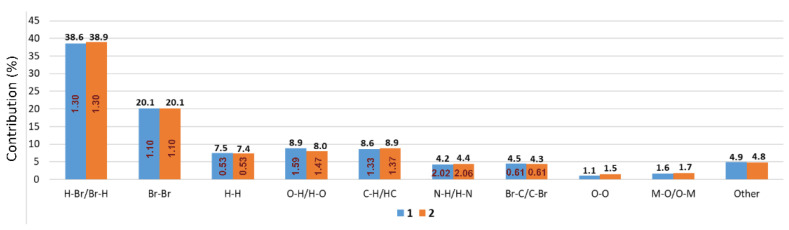
Percentage contribution of particular interatomic contacts to the Hirshfeld Surface areas for 1 (blue) and 2 (orange). The chart is decorated with respective enrichment ratio (ER) values (in red). ER values were not computed for actual contacts contribution below 2% and random contacts below 1% since they are not meaningful.

The decomposition of FPs (Figure 10) shows that H⋯Br contacts comprise 38.6% (in **1**) and 38.9% (in 2) of the total HS area. Moreover, the enrichment ratio values (*ER_HBr_* = 1.30) are higher than unity, which suggests that this halogen bonding may play a key role in the supramolecular assembly of polymeric chains.

Similarly, Br⋯Br contacts are crucial for crystal packing description. These contacts reach 20.1% in the studied structures and their ER values *ER_BrBr_* = 1.10 also suggest that these are favored. Both types of interactions, i.e., H⋯Br and Br⋯Br associate neighboring polymeric chains within the crystal structure. H^...^H contacts comprising 7.4–7.5% mainly follow the intramolecular steric interactions between C–H groups of aromatic fragments. However, their ERs are significantly lower than unity, which indicates that these contacts are disfavored in this structure. In contrast, electrostatic O⋯H, C⋯H, and N⋯H contacts are underrepresented since their ERs are higher than unity in both structures. The presence of C⋯H contacts follows the π-facial hydrogen bonds, while strongly favored (ER_NH_ = 2.02 and ER_NH_ = 2.06 in 1 and 2, respectively). N⋯H contacts are caused mostly by O–H⋯N interactions between polymeric chains. Even though, in both structures, Br⋯C contacts cover about 5% of the HS surfaces, their ERs suggest that they are also disfavored.

## 4. Conclusions

Studied crystal systems 1 and 2 are isostructural. MS data for La(III) and Sm(III) complexes detected several profiles of ion currents. Emission of gaseous products in particular steps of thermal decomposition of La(III) and Sm(III) complexes corresponds with mass losses on TG curves. The central atom replacement resulted in unit cell identity parameter П = 0.0091. The value close to 0 indicates great similarity of the compared unit cells. Moreover, structural studies augmented by Hirshfeld Surface analysis of coordination polymers 1 and 2 clearly confirmed their isostructurality. Both supramolecular systems are characterized by almost identical HS and FP shapes, which follow the identity of their assembly. Percentage contributions of particular interatomic contacts to the HSs revealed that H⋯Br and Br⋯Br contacts play a crucial role in the stabilization of crystal systems of 1 and 2. However, despite relatively low percentage contributions, other electrostatics as C⋯H, O⋯H, and N⋯H are also significant for the assemblies of these isostructural systems. The O–H^...^O (Br) hydrogen bonds are responsible for the stabilization of the coordination sphere, while the O–H^...^N bonds are responsible for the formation of a layered system (ladder). Research on such structures should be continued due to the broad possible applications of polymeric coordination compounds, e.g., in medicine, absorption, catalysis, sensors, and electronic devices. The obtained compounds represent a remarkable and promising field of chemistry and, therefore, research on this type of coordination compound should be continued.

## Figures and Tables

**Table 1:** Thermal decomposition data of La(III) and Sm(III) compounds.

Compound	Range of Decomposition/°C	Mass Loss/%		Intermediate and Residue Solid Products
		Found	Calc.	
La_2_(4,4′-bpy)_1.5_(CBr_2_HCOO)_6_(H_2_O)_2_	50–135	2.0	1.95	La_2_(4,4′-bpy)_1.5_(CBr_2_HCOO)_6_
	200–340	34.0	33.84	La_2_(4,4′-bpy)(CBr_2_HCOO)_2_Br_4_
	340–950	47.0	46.59	La_2_O_3_
Sm_2_(4,4′-bpy)_1.5_(CBr_2_HCOO)_6_(H_2_O)_2_	50–125	2.0	1.92	Sm_2_(4,4′-bpy)_1.5_(CBr_2_HCOO)_6_
	195–325	33.5	33.43	Sm_2_(4,4′-bpy)(CBr_2_HCOO)_2_Br_4_
	325–945	45.5	46.02	Sm_2_O_3_

**Table 2:** Crystal and structure refinement data for study complexes.

Crystal Data		
Chemical formula	C_27_H_22_Br_12_N_3_O_14_ La_2_ (1)	C_27_H_22_Br_12_N_3_O_14_Sm_2_ (2)
*M*_r_	1849.21	1872.09
Crystal system, space group	Triclinic, *P*-1	Triclinic, *P*-1
Temperature (K)	296	296
*a*, *b*, *c* (Å)	9.6064 (4), 11.0105 (5), 22.9742 (9)	9.3646 (3), 11.0114 (4), 22.7802 (8)
α, β, γ (°)	82.468 (4), 87.786 (3), 85.730 (4)	82.242 (3), 88.230 (3), 86.388 (3)
*V* (Å^3^)	2401.33 (18)	2322.32 (14)
*Z*	2	2
Radiation type	Mo *K*α	Mo *K*α
μ (mm^−1^)	11.80	12.89
Crystal size (mm)	0.3 × 0.3 × 0.1	0.4 × 0.2 × 0.1
**Data Collection**		
Diffractometer	Kuma KM-4 CCD	Kuma KM-4 CCD
Absorption correction	Multi-scan *CrysAlis RED*, Oxford Diffraction Ltd., Version 1.171.33.66 (release 28-04-2010 CrysAlis171.NET) (compiled Apr 28 2010,14:27:37) Empirical absorption correction using spherical harmonics, implemented in SCALE3 ABSPACK scaling algorithm.	Multi-scan *CrysAlis PRO* 1.171.41.76a (Rigaku Oxford Diffraction, 2020) Empirical absorption correction using spherical harmonics, implemented in SCALE3 ABSPACK scaling algorithm.
*T*_min_, *T*_max_	0.250, 1.000	0.106, 1.000
No. of measured, independent and observed [*I* > 2σ(*I*)] reflections	33071, 8797, 6405	32238, 8811, 7090
*R*_int_	0.034	0.053
(sin θ/λ)_max_ (Å^−1^)	0.602	0.610
Refinement		
*R*[*F*^2^ > 2σ(*F*^2^)], *wR*(*F*^2^), *S*	0.031, 0.073, 1.00	0.047, 0.114, 1.02
No. of reflections	8797	8811
No. of parameters	609	586
No. of restraints	258	234
H-atom treatment	H atoms treated by a mixture of independent and constrained refinement	H atoms treated by a mixture of independent and constrained refinement

**Table 3:** Hydrogen-bond geometry (Å, º) for compound 1.

*D*—H···*A*	*D*—H	H···*A*	*D*···*A*	*D*—H···*A*	Symmetry Codes	Graph-Set
O1W—H1WA···Br5C	0.83 (1)	2.95 (4)	3.589 (4)	135 (5)		R (7)
O1W—H1WB···N1^i^	0.84 (1)	2.84 (7)	3.231 (6)	110 (6)	(i) *x* + 1, *y*, *z*	R (8)
O1W—H1WB···O2B^i^	0.83 (1)	2.07 (3)	2.812 (5)	147 (5)	(i) *x* + 1, *y*, *z*	R (6)
O2W—H2WA···O1E^ii^	0.84 (1)	2.16 (3)	2.907 (5)	148 (5)	(ii) *x* − 1, *y*, *z*	R (6)
O2W—H2WA···O1B	0.84 (1)	2.94 (7)	3.304 (6)	109 (6)		R (6)
O2W—H2WB···N20^iii^	0.84 (1)	1.90 (1)	2.734 (6)	173 (5)	(iii) −*x*, −*y* + 2, −*z* + 2	C (22)

**Table 4:** Hydrogen-bond geometry (Å, º) for compound 2.

*D*—H···*A*	*D*—H	H···*A*	*D*···*A*	*D*—H···*A*	Symmetry Codes	Graph-Set
O1W—H1WA···Br5C	0.84 (1)	2.98 (7)	3.566 (6)	129 (8)		R (7)
O1W—H1WB···N1^i^	0.84 (1)	2.49 (5)	3.164 (8)	139 (6)	(i) *x* + 1, *y*, *z*	R (8)
O1W—H1WB···O2B^i^	0.84 (1)	2.07 (6)	2.757 (7)	138 (8)	(i) *x* + 1, *y*, *z*	R (6)
O2W—H2WA···O1B	0.84 (1)	2.62 (7)	3.270 (7)	135 (8)		R (6)
O2W—H2WA···O1E^ii^	0.84 (1)	2.21 (6)	2.862 (7)	135 (7)	(ii) *x* − 1, *y*, *z*	R (6)
O2W—H2WB···N20^iii^	0.84 (1)	1.92 (2)	2.745 (9)	168 (8)	(iii) −*x*, −*y* + 2, −*z* + 2	C (22)

## Supplementary Materials

2021532 and 2021533 contain the supplementary crystallographic data for this paper. The data is provided free of charge by The Cambridge Crystallographic Data Centre via www.ccdc.cam.ac.uk/structures.
